# Longitudinal SARS-CoV-2 antibody response in a healthcare worker cohort utilising the Abbott Alinity® anti-nucleocapsid assay

**DOI:** 10.1371/journal.pone.0325544

**Published:** 2025-06-11

**Authors:** Stephen P. Connolly, Alejandro Garcia Leon, Sandra Green, Daragh McGee, Paul Duggan, Robert Browne, Sarah Miles, Riya Negi, Dana Alalwan, Dominick Natin, Patrick W. Mallon, Peter O’Gorman, Graham Lee, David Green, Tara McGinty, Aoife G. Cotter

**Affiliations:** 1 Department of Infectious Diseases, Mater Misericordiae University Hospital, Dublin, Ireland; 2 School of Medicine, University College Dublin (UCD), Dublin, Ireland; 3 Centre for Experimental Pathogen-Host Research (CEPHR), University College Dublin (UCD), Dublin, Ireland; 4 Department of Occupational Medicine, Mater Misericordiae University Hospital, Dublin, Ireland; 5 Department of Pathology, Mater Misericordiae University Hospital, Dublin, Ireland; 6 Department of Clinical Biochemistry, Mater Misericordiae University Hospital, Dublin, Ireland; University of the Witwatersrand, SOUTH AFRICA

## Abstract

**Introduction:**

Healthcare workers (HCWs) in Ireland bore a particularly high burden of SARS-CoV-2 infections, representing over 30% of infections during initial waves. We describe the prevalence, incidence and persistence of SARS-CoV-2 anti-nucleocapsid (anti-NC) IgG positivity in a cohort of HCWs working in a Dublin inner-city tertiary hospital, over 48 weeks.

**Methods:**

The SORTeD (Seroprevalence, Seroconversion Rates and Transmission Dynamics of SARS-CoV-2 among Healthcare Workers) study was a longitudinal cohort study of HCWs working in an inner-city hospital in Dublin between July 2020 and September 2021. Participants had either a prior history of PCR-confirmed SARS-CoV-2 (Group 1) or no prior history of SARS-CoV-2 (Group 2). Serum samples were obtained at weeks 0, 12 and 48, and tested for SARS-CoV-2 nucleocapsid (NC) antibody using a qualitative immunoassay (Abbott Alinity®). Seroprevalence rates are presented using descriptive statistics, with univariate and multivariate analysis examining associations between participant characteristics, IgG status and refractive index in Group 1. Data is presented as n (%) or median (interquartile range (IQR)) where appropriate.

**Results:**

Of the 395 HCWs who were recruited, 304 (77.0%) were female, median age was 33 (28−45) years, and 343 (86.8%) had patient-facing roles. In Group 1, time from infection to sampling was 173 (144.0–202.0) days. Seroprevalence of IgG in Group 1 at 0, 12 and 48 weeks was 47.4%, 19.0% and 7.3%, respectively; while seroprevalence in Group 2 was 5.4%, 4.3% and 2.6%, respectively. A lower refractive index was seen in higher sampling intervals (r = −0.5, 95% CI −0.576 to −0.427; p < 0.001). Fourteen incident infections were reported by the cohort during the study, and 3 documented reinfections.

**Conclusion:**

Our study shows low seroprevalence in prior confirmed cases among our HCW population, possibly explained by reduced sensitivity of this assay with increasing time from SARS-CoV-2 exposure and timing of testing. Confirmatory testing with a quantitative assay would help understand the true seroprevalence of SARS-CoV-2 IgG in this cohort.

## Introduction

Since the arrival of SARS-CoV-2 in Ireland in late February 2020, over 1.8 million infections have been identified, with the virus implicated in almost 10,000 deaths to date [1]. Ireland’s healthcare workers (HCWs) bore a particularly high burden of SARS-CoV-2 infections in the initial phase of the virus’s spread, representing over 30% of all confirmed infections in Ireland until late August [2]. Mandatory quarantine for both confirmed cases and close contacts of confirmed cases served to deplete the health system’s workforce.

Seroprevalence studies have utility in detailing which sectors of our HCW workforce were affected and help inform infection prevention and control measures in the workplace in the future. Symptom-directed, reverse transcription polymerase chain reaction (RT-PCR) testing for acute SARS-CoV-2 infections under-represents the extent of exposure in a population, given the well-documented phenomenon of asymptomatic infection [3]. Serological testing allows us to describe the proportion of the population with prior exposure, including asymptomatic infection. The “Study to Investigate COVID-19 Infection in People Living in Ireland” (SCOPI Study) conducted antibody testing on a group of invited participants selected at random from two Irish counties with contrasting cases rates. It found that among the 1.7% of those tested with detectable antibodies directed at SARS-CoV-2, 33% did not recall symptoms consistent with COVID-19 [4].

Finally, duration of immunity remains an area of intense research; specifically in those with a measurable antibody response, both how long that response lasts and the factors associated with durability and the nature of reinfection are under evaluation.

The primary objectives of the SORTeD (Seroprevalence, Seroconversion Rates and Transmission Dynamics of SARS-CoV-2 among Healthcare Workers) study were to determine:

(i) The prevalence of SARS-CoV-2 IgG in our institution’s HCW population across two groups
  a Those with prior SARS-CoV-2 infection (confirmed via molecular testing) at baseline (Group 1).
  b Those without prior, confirmed SARS-CoV-2 (Group 2).
(ii) The duration of the persistence of detectable IgG to SARS-CoV-2 in those with detectable SARS-CoV-2 IgG
(iii) The seroconversion rate of those with no prior history of SARS-CoV-2 infection over 48 weeks

The secondary objective was to explore the occupational risk factors and host factors associated with higher likelihood of SARS-CoV-2 seropositivity in our cohort. It was hypothesised that seroprevalence among Group 1 would be higher than Group 2 and that IgG seropositivity would be associated with fewer incident infections.

## Methods

### Study design and population

The SORTeD project was a prospective, single site, observational cohort study of HCWs working in Mater Misericordiae University Hospital (MMUH), Dublin, as part of collaboration between the departments of infectious diseases, occupational health, and biochemistry. Staff members both with and without a history of prior SARS-CoV-2 infection were eligible for enrolment. Participants were assigned to one of two groups:

(i) Those with a confirmed prior acute SARS-CoV-2 infection via RT-PCR (Group 1).
(ii) Those without a prior diagnosis of SARS-CoV-2 infection (Group 2).

Participants were required to be staff members, be aged 18 years or over, and be expected to remain in their post for the duration of the study.

### Study assessments

Between the 13^th^ of August and the 2^nd^ of December 2020, staff members of MMUH were recruited through a combination of internal advertisements and emailed invitations to staff members known to MMUH infectious diseases and occupational health departments. Following written, informed and witnessed consent; participants were asked to provide blood samples and to complete paper questionnaires detailing demographic details, details surrounding SARS-CoV-2 vaccination (i.e., model received and date), severity of their index COVID-19 illness (if any) and the presence of confirmed or suspected SARS-CoV-2 reinfection. Efforts were made to recruit from a large cross-section of departments to reduce the impact of selection bias. In Group 2, participants were asked to recall symptoms since March 2020 that conceivably could have related to COVID-19, such as fever, myalgia and cough. As widespread SARS-CoV-2 vaccination was implemented in January 2021, questionnaires were modified to collect details surrounding vaccination status, i.e., the timing and vaccine model administered to each participant. Participant visits were repeated at 12 and 48 weeks. Collected samples were tested for SARS-CoV-2 IgG on the Abbott Alinity® testing platform (Abbott Laboratories, Chicago, Illinois, USA), a qualitative chemiluminescence assay (CMIA) which classifies results as ‘detectable’ if the obtained refractive index was ≥1.4 (as per the manufacturer’s guidelines). Significant delays existed in sourcing a serological assay in Ireland, and at the time of devising the protocol for the SORTeD study, this was the only available assay to our study group. Participants’ data was stored in a pseudonymised form with the key to identifying participants held securely by the lead author.

### Statistical analyses

Based on limited international data at the time of the study’s conception, an assumption of a 25% prevalence of SARS-CoV-2 IgG among HCWs was made. This, along with a desired 95% level of confidence and a 5% margin of error, required that a minimum of 184 HWCs were recruited to each group to estimate the prevalence of SARS-CoV-2 among MMUH HCWs, with a targeted amount of 200 to each group to mitigate loss to follow-up. Incident cases during the study were defined as SARS-CoV-2 cases confirmed by RT-PCR reported either by self-report or confirmed with occupational health records. Missing data points, including results from those lost to follow up, were excluded from each analysis rather than estimated, and were assumed to be missing at random. Simple descriptive statistics were used to describe demographics, the frequencies of certain baseline characteristics of the participants, and prevalence of detectable IgG directed against SARS-CoV-2 as a whole and within each group. Univariate analysis was performed using Chi-squared or Fischer’s exact test for categorical variables with respect to their influence on the binary outcome of IgG seropositivity. Odds ratios (ORs) with 95% confidence intervals (CIs) were calculated to convey the relative odds of seropositivity in certain groups. Mann-Whitney U test or Spearman’s correlation were used for continuous variables (i.e., sampling interval and refractive index). Following this, multiple logistic regression analysis was conducted to control for potential confounding factors and to further evaluate the independent predictors of IgG seropositivity. All analyses were performed using IBM SPSS Statistics (versions 26.0 and 27.0 for Windows, IBM SPSS Inc, Chicago, Illinois, USA). All graphs, including bar charts and scatter plots, were created using GraphPad Prism (version 9.3.1 for Windows, GraphPad Software, Boston, Massachusetts, USA). Data are presented as n (%) or median (IQR), where appropriate. p < 0.05 was considered statistically significant.

### Ethical approval

Ethical approval for the study was obtained from the Mater Misericordiae University Hospital Institutional Review Board (reference 1/378/2165). All HCWs participated with individual, written and informed consent for samples and data to be collected, stored, analysed and published in aggregate form.

## Results

### Baseline characteristics of the SORTeD cohort

Three-hundred and ninety-five participants were recruited, with 192 and 203 participants assorted to Groups 1 and 2, respectively. All applicants were deemed eligible for inclusion. Assuming the staff number to be the overall reported figure of 3000 members, this comprises approximately 13% of the hospital’s workforce. Demographic data are presented in Table 1. The staff division with greatest representation was nursing, comprising 42% of the overall cohort. Group 1 had a greater proportion of nursing or healthcare assistant staff while Group 2 contained more allied health or radiology staff. A lesser proportion of Group 1 were Caucasian compared to Group 2 (79.2% versus 91.1%; p < 0.001). Of the 53 participants not identifying as Caucasian; 46 (86.8%) identified as being of Asian origin, 4 participants (7.5%) identified as being of African origin, and 3 participants (5.7%) identified as being of South American origin.

**Table 1:** Baseline characteristics of Groups 1 and 2 of the SORTeD cohort (N.B. “sampling interval in Group 2 pertains to those who reported prior symptoms compatible with COVID-19).

	Overall cohort	Group 1	Group 2	Between- Group p value
	n (%)	n (%)	n (%)	
	(n = 395)	(n = 192)	(n = 203)	
**Demographics**				
Female	304 (77)	145 (75.5)	159 (78.3)	0.62
Caucasian	337 (85.3)	152 (79.2)	185 (91.1)	<0.01
Median age (IQR^a^)	33 (45−28)	37 (49−29)	32 (42−28)	0.01
Current or ex-smoker	57 (14.4)	22 (11.5)	35 (17.2)	0.88
**Co-morbid conditions**				
Cardiovascular disease (“heart disease”)	6 (1.5)	4 (2.1)	2 (1.0)	0.024
Chronic lung disease or asthma	34 (8.6)	21 (10.9)	13 (6.4)	0.081
Diabetes mellitus (type I or II)	5 (1.3)	3 (1.6)	2 (1.0)	0.734
Systemic immunosuppressants	6 (1.5)	4 (2.1)	2 (1.0)	0.009
**Patient exposure**				
Patient-facing	343 (86.8)	174 (90.6)	169 (83.3)	<0.01
Registered nurse	166 (42.0)	90 (46.9)	76 (37.4)	0.02
Medical doctor	70 (17.7)	33 (17.2)	37 (18.2)	0.46
Other non-clinical staff	58 (14.7)	29 (15.1)	29 (14.3)	0.46
Healthcare Assistant	19 (4.8)	15 (7.8)	4 (2.0)	<0.01
Allied Health	40 (10.1)	13 (6.8)	27 (13.3)	0.02
Radiology/x-ray technician	35 (8.9)	9 (4.7)	26 (12.8)	<0.01
COVID ward	112 (28.3)	53 (27.6)	59 (29.1)	0.36
Non-COVID ward	198 (50.1)	116 (60.4)	82 (40.4)	<0.01
ICU^b^/HDU^c^	73 (18.5)	28 (14.6)	45 (22.2)	0.05
ED^d^	70 (17.7)	25 (13.0)	45 (22.2)	0.01
Sampling interval from symptoms to baseline visit [(median (IQR)) days]	173 (202.0-144.0)	186 (155.5-210.0)	150 (80.0-173.5)	<0.01

Legend:

^a^Interquartile range,

^b^intensive care unit,

^c^high-dependency unit,

^d^emergency department.

Of the 192 participants enrolled in Group 1, 183 of participants (95%) described prior symptoms, with 151 (78.6%) reporting respiratory symptoms (cough, dyspnoea, sore throat or coryza), while 129 (67.2%) reported anosmia. Nineteen participants (9.9%) required hospital admission, with a median length of stay of 3 (1.5–5.0) days. Seventy-eight participants within Group 2 (38.4%) reported prior respiratory symptoms, i.e., dyspnoea, cough or coryza since March 1^st^ 2020. The common reported individual symptoms were headache (58 (28.6%)), fatigue (49 (24.1%)), sore throat (45 (22.2%)) and coryza (35 (17.2%)).

### Baseline seroprevalence

Baseline prevalence of IgG directed against the SARS-CoV-2 NC antigen was 47.4% and 5.4% in Groups 1 and 2, respectively (Table 2), and the IgG refractive index was 1.23 (3.11–0.47) and 0.03 (0.070–0.02) respectively. The median sampling interval (days between study visit and symptom onset) in those with reported symptoms was greater in Group 1 (186 days; (155.5–210.0)) than in those reporting symptoms in Group 2 (150 days (80.0–173.5) p < 0.01). Of the 11 participants in Group 2 with detectable IgG, 8 (72.7%) reported prior respiratory symptoms.

**Table 2:** Longitudinal serology results, with retention within SORTeD cohort at weeks 0, 12 and 48.

Entire Cohort		395 (100)	
	Visit 1 (Week 0)	Visit 2 (Week 12)	Visit 3 (Week 48)
Retention within the study n (%)	395 (100)	340 (86)	276 (70)
**Group 1**			
IgG detected n (%)	91 (47.4)	29 (19.00)	9 (7.30)
IgG refractive index (median (IQR))	1.23 (0.47-3.11)	0.37 (0.10-1.12)	0.15 (0.06-0.44)
Total samples analysed (n)	192	153	124
**Group 2**			
IgG detected n (%)	11 (5.40)	8 (4.30)	4 (2.60)
IgG refractive index (median (IQR))	0.03 (0.02-0.07)	0.03 (0.02-0.09)	0.02 (0.01-0.07)
Total samples analysed (n)	203	187	152

Legend: ^a^Interquartile range.

### Longitudinal follow up

**Fig 1:**
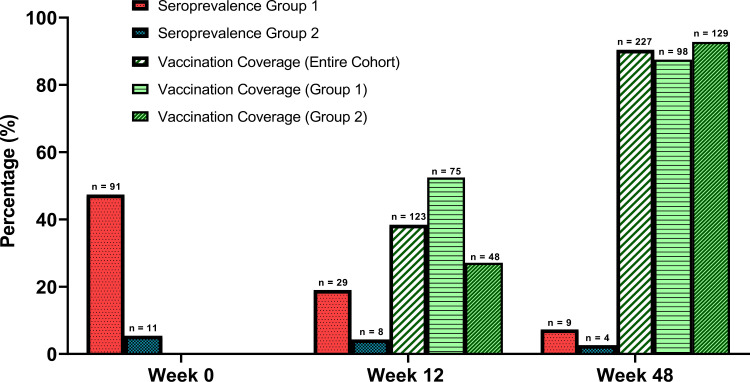
Seropositivity and vaccination of the cohort during the study. Bar charts showing the absolute numbers proportions (%) of participants testing positive for SARS-CoV-2 IgG at weeks 0, 12, and 48; as well as proportions who were fully vaccinated (defined as having had their seconds dose ≥ 7 days) at each time point.

Two hundred and seventy (68.4%) of the original 395 participants completed the final visit of the study. Overall serology results and retention in the study are displayed in Table 2. The numbers and proportions of those testing positive for SARS-CoV-2 IgG are displayed in Fig 1. Of the 102 patients testing with detectable IgG at baseline (n = 91 in Group 1, n = 11 in Group 2), only 33 were detectable at week 12 (n = 28 in Group 1, n = 5 in Group 2), while only 9 (8.8%) were detectable by week 48 (n = 8 in Group 1, n = 1 in Group 2). IgG seroprevalence fell to 7.3% in Group 1 by week 48, and 2.6% in Group 2. Between weeks 0 and 48, Group 1, saw a fall in seropositivity of 84.6%, or 1.7% per week. Group 2 saw a reduction of 51.9%, or 1.1% per week. Among those with initially detectable IgG results in Group 1, the median refractive index at the week 0, week 12 and week 48 visits was 3.13 (2.07–5.04), 1.12 (0.52–2.16), and 0.29 (0.16–0.83), respectively. By week 48, refractive index had fallen by a median of 2.75 (1.66–3.34) in these participants (S1 Fig).

As indicated in Fig 1, vaccine coverage (defined here as having had a second dose of a SARS-CoV-2 vaccine of any form ≥ 7 days prior to blood sampling) rose from 0.0% at baseline to 38.4% at week 12 and 90.4% at week 48. By week 48, the most frequent administered vaccine was the Pfizer/BioNtech (BNT162b2) at 91.7%, followed by the Astra Zeneca (ChAdOx1-S), which comprised of 8.3% of all vaccines administered.

### Incident infection

Fourteen incident infections confirmed via RT-PCR were reported by the cohort during their enrollment in the study (6 in Group 1, 8 in Group 2), including 3 documented reinfections. Of the 293 participants with follow up samples who tested negative initially, 4 participants subsequently seroconverted by week 12 (3 in Group 2 and 1 in Group 1) with only 2 of these participants recalling symptoms (S1 Table). Only 8 of the patients who had a detectable IgG at baseline continued to have a detectable IgG at week 48.

By week 48, only 3 of the cohort of Group 2 testing initially negative had detectable IgG, amounting to one participant acquiring the infection per 16 week period. None of this group reported new symptoms in the intervening months. The changes in proportions of positive results are displayed in Fig 1.

### Influence of sampling interval

When all IgG results across all study visits in the Group 1 (i.e., those with prior symptoms and confirmed prior COVID-19) were compared to their respective sampling intervals, median sampling interval (i.e., interval from symptoms onset to blood test) was significantly lower in the those with positive results (190 days; IQR 155.0–253.0) versus negative results (328.0 days; IQR 212.0–482.0; p < 0.001). Greater sampling interval was associated with less likelihood of seropositivity (OR 0.983 per additional day, 95% CI 0.98–0.99) and the sampling interval correlated inversely with the IgG refractive index (r = −0.5; 95% CI −0.58 to −0.43; p < 0.001).

### Correlates of seropositivity

In those with prior confirmed SARS-CoV-2 infection, IgG seropositivity was less likely in non-Caucasian participants (OR 0.37, 95% CI 0.18–0.78; p = 0.01), and more likely in those requiring admission to hospital during the index illness (OR 3.5, 95% CI 1.20–10.17, p = 0.02), and those deployed to the ICU or HDU (OR 1.53, 95% CI 0.88–2.65; p = 0.13). Increasing age was associated with increased odds of seropositivity, with an OR of seropositivity of 2.59 (95% CI 1.64–4.13; p < 0.001) in those older than 40 years, and an OR of 1.01 per year older, (95% CI 1.010–1.082; p = 0.08. No correlation between seropositivity and individual co-morbid conditions was seen (S2 Table).

## Discussion

Our study describes the seroprevalence of SARS-CoV-2 anti-nucleocapsid IgG in our cohort, at baseline and at two additional time points over 48 weeks. We show that 47.4% of participants with prior, PCR-confirmed SARS-CoV-2 infection had a detectable IgG when measured, with a low prevalence of antibodies at 5.4% of the asymptomatic/control group. Our cohort demonstrated a reduction of seroprevalence for anti-nucleocapsid IgG in Group 1 from 47.4% to 7.3% over 48 weeks, and a reduction from 5.4% to 2.6% in Group 2. The other primary aim of the study was to describe the seroconversion rate of previously SARS-CoV-2 non-infected participants over 48 weeks. This amounted to one person seroconverting from Group 2 per 16-week period, which is surprisingly low.

A higher proportion of detectable antibody at baseline in Group 1 was noted in non-Caucasian participants and those of higher age. Greater and longer-lasting antibody responses in non-white or non-Caucasian groups have been reported in number of studies examining the response to both SARS-CoV-2 vaccination and natural infection [5–7]. It has been recognised in a number of studies that COVID-19 case mortality, even when matched for age, is greater for such populations [8,9]. A number of potential confounding pathophysiological and socioeconomic factors can potentially explain this however, although disease severity is thought to correlate with anti-SARS-CoV-2 IgG titres [10]. This is consistent with the findings of our study, which showed higher odds of seropositivity at baseline among those requiring admission, and a higher median refractive index in those with dyspnoea and the need for admission. Increasing age has been associated with both higher and lower magnitudes of humoral response in response to both SARS-CoV-2 infection and vaccination, with systematic reviews hampered by inconsistent adjustment for the potential confounding influence of disease severity [11–14].

While 38.4% of the patients in Group 2 reported recent respiratory symptoms consistent with COVID-19, only 5.4% of the cohort tested positive for IgG. This questions the reliability of recalled symptoms for determining prior COVID-19 in populations, or is a function of our assay’s sensitivity. In addition, 81.8% of those detecting positive for SARS-CoV-2 IgG in Group 2 recalled respiratory symptoms, a figure comparable to other published studies [4,15,16].

The incidence of SARS-CoV-2 seroconversion and new diagnoses of acute SARS-CoV-2 infection were relatively low in our cohort, and a variety of factors potentially contributed to this. Published data, including a number of systematic reviews, indicates that public health measure such as social distancing, hand hygiene and “lockdown” measures served to reduce the incidence of SARS-CoV-2 infection in the earlier phases of the pandemic and throughout the duration of our study [17,18]. The benefit of use personal protective equipment (PPE) in the healthcare setting with respect to SARS-CoV-2 transmission is also well documented [19]. Our institution was fortunate in having a ready supply of PPE for staff members, with training and mandatory use of gowning, gloves and filtering facepiece (FFP) masks in the care of all patients with confirmed on suspected SARS-CoV-2 infection. Use of surgical masks became mandatory for HCWs at all times before the study commenced, and continued for its duration. Strict isolation at home was enforced for all staff members with respiratory symptoms while pending PCR testing via occupational health, however asymptomatic testing via PCR of HCWs was not generally practiced. Published evidence suggests that in the initial waves of COVID-19 at least, SARS-CoV-2 vaccination appeared to not only reduce disease severity but also attenuate the incidence of infection [20]. The introduction of widespread vaccination for SARS-CoV-2 that commenced during the study period, in combination with the other public health and infection control measures outlined above, is therefore likely to have reduced the number of incident infections in our cohort. Adapting to these rapid changes in the scientific landscape is one of the main challenges however in performing a hospital-wide epidemiological study during a rapidly-evolving pandemic.

In addition to a lower than expected rate of seropositivity in those with prior infection at baseline, our data also show a large decline in both the proportions of detectable IgG results and refractive indices over time. A variety of potential explanations exist for this. While lesser magnitudes of humoral responses to SARS-CoV-2 are reported in those with several co-morbid conditions or taking immunosuppressant medications, relatively few of our cohort reported any history of chronic disease [11,13]. The high median sampling intervals between disease onset (in those with prior SARS-CoV-2 infection) in our cohort is another explanation. Reduced sensitivity of both the Abbott Alinity and Architect assays with increasing time has been reported in a number of longitudinal studies of cohorts of prior PCR-positive patients in the similar Abbott Architect anti-NC IgG assay [21–23]. Other data indicate that not only is the Abbott Architect/Alinity, but also that anti-NC as a target in general, may be associated with lower detection rates. Heffernan et al.’s Irish-based study assessing the sensitivity and specificity of four differing ELISA assays of varying targets and manufacturers, finding the lowest sensitivity at > 14 days among the anti-NC assays [24]. This questions the applicability of the assay for other cross-sectional and longitudinal serostudies, particularly as the pandemic matures.

While seroprevalence for SARS-CoV-2 was, by definition, constantly changing during the early waves of COVID-19, therefore limiting comparison with studies of other populations taken at different points of the pandemic, the cross-sectional results for Group 2 in our study are comparable to other published seroprevalence studies of SARS-CoV-2 IgG in the Irish general and HCW population, such as the SCOPI study, which found a background seroprevalence of 3.1% among the general population in the Dublin area in June and July 2020. It is also comparable to the seroprevalence found during October 2020 in the Galway centre of the PRECISE study (4.1%), and higher than that found in the asymptomatic arm of Faller et al.’s Cork-based study, where samples were taken between May and June 2020 [4,16,25,26]. While too numerous to scrutinise in their entirety, a number of international serological studies assessing seroprevalence in the general population and HCWs were compared to our results. As was for the Irish studies, heterogeneity between the assays used and the time point in the pandemic at which they were conducted, as well as differences in vaccination rates and public health measures employed in each location, make their comparison difficult.

This study has the advantage of collecting a rich dataset of a cohort of healthcare workers over multiple time points in a single institution, across a broad array of disciplines, during a challenging time in the history of Ireland’s healthcare. It succeeded in its primary aim to describe the prevalence of seropositivity in a cohort of HCWs with and without prior history of PCR-confirmed SARS-CoV-2 infection, as well as describing the rate of incident infection among the Group 2. The study was limited by the low positivity rate, which hampered the analysis of the secondary outcomes by virtue of the original calculations of study size assuming IgG prevalence of 25% among the cohort. These numbers proved to be inadequate to detect meaningful numbers of reinfections. As a result of doubts about the durability of antibody detectability in follow up with our selected assay, the true seroconversion rate has likely not been fully ascertained. Interpretation of magnitude of refractive indices exceeding the cut-off in qualitative chemiluminescence assays as a surrogate of antibody titre is discouraged, and linear relationships with antibody titres have not been reliably shown. The relatively low proportion of non-Caucasian participants signing up to the study (11.6% overall) also limits the generalisability of these findings to those of other racial origins. Finally, the practice of advertising research studies in order to recruit volunteer participants is at inherent risk of bias, given participants with recalled symptoms who were not originally eligible for RT-PCR testing under the original criteria could conceivably enrol to determine the cause of their recalled illness.

## Conclusion

Our study shows declining positivity rates for SARS-CoV-2 anti-nucleocapsid IgG in those previously infected with the virus. Unexpectedly low rates of positive IgG occurred in both groups in follow up, with fewer participants testing positive in Group 2 at week 48 than at baseline. The potential reasons for this include low incidence of natural infection attributable largely to the advent of SARS-CoV-2 vaccination and other concurrent public health measures, as well as potential issues regarding the sensitivity of assay when the interval from initial infection and blood sampling are large. Confirmatory, quantitative antibody testing is more likely to assist in discerning true seroprevalence and seroconversion rates.

## Supporting information

S1 Fig Refractive index dynamics of those testing initially positive for SARS-CoV-2 at each time point in the study period; via (a) scatter plot with medians indicated via horizontal line, and (b) via spaghetti plot. (PDF)

S2 Fig Poster for European Congress of Medical Microbiology and Infectious Diseases 2023, Copenhagen, Denmark. (PDF)

S1 Table Incident infection in the cohort during the study period. Legend: ^a^reverse transcription polymerase chain reaction.
(DOCX)

S2 Table Correlates of seropositivity in the cohort. Legend: ^a^odds ratio, ^b^confidence interval, ^c^high-dependency unit, ^d^intensive care unit.
(DOCX)
