# Supplementary figures and images for: Longitudinal SARS-CoV-2 antibody response in a healthcare worker cohort utilising the Abbott Alinity® anti-nucleocapsid assay
Source: PLoS One. 2025 Jun 11;20(6):e0325544. doi: 10.1371/journal.pone.0325544 (PMC12157052; doi:10.1371/journal.pone.0325544)

(a)

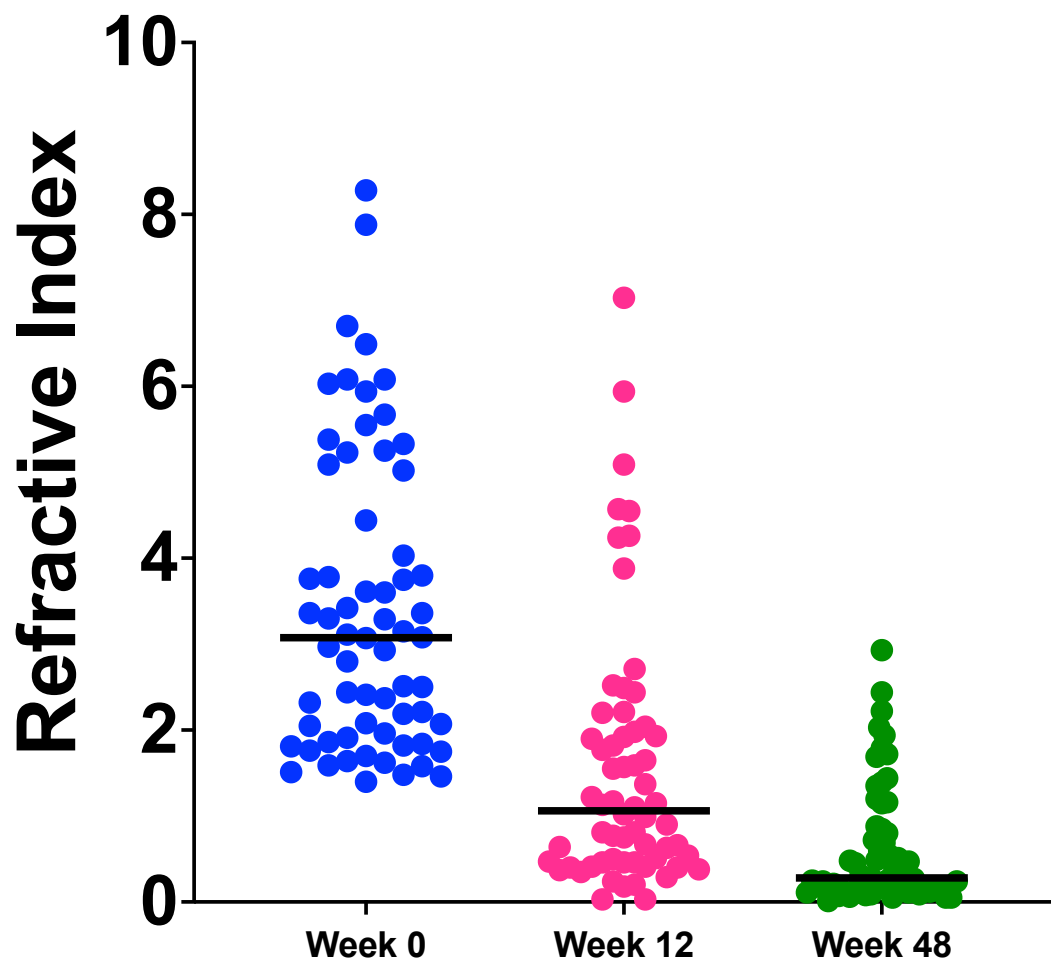

(b)

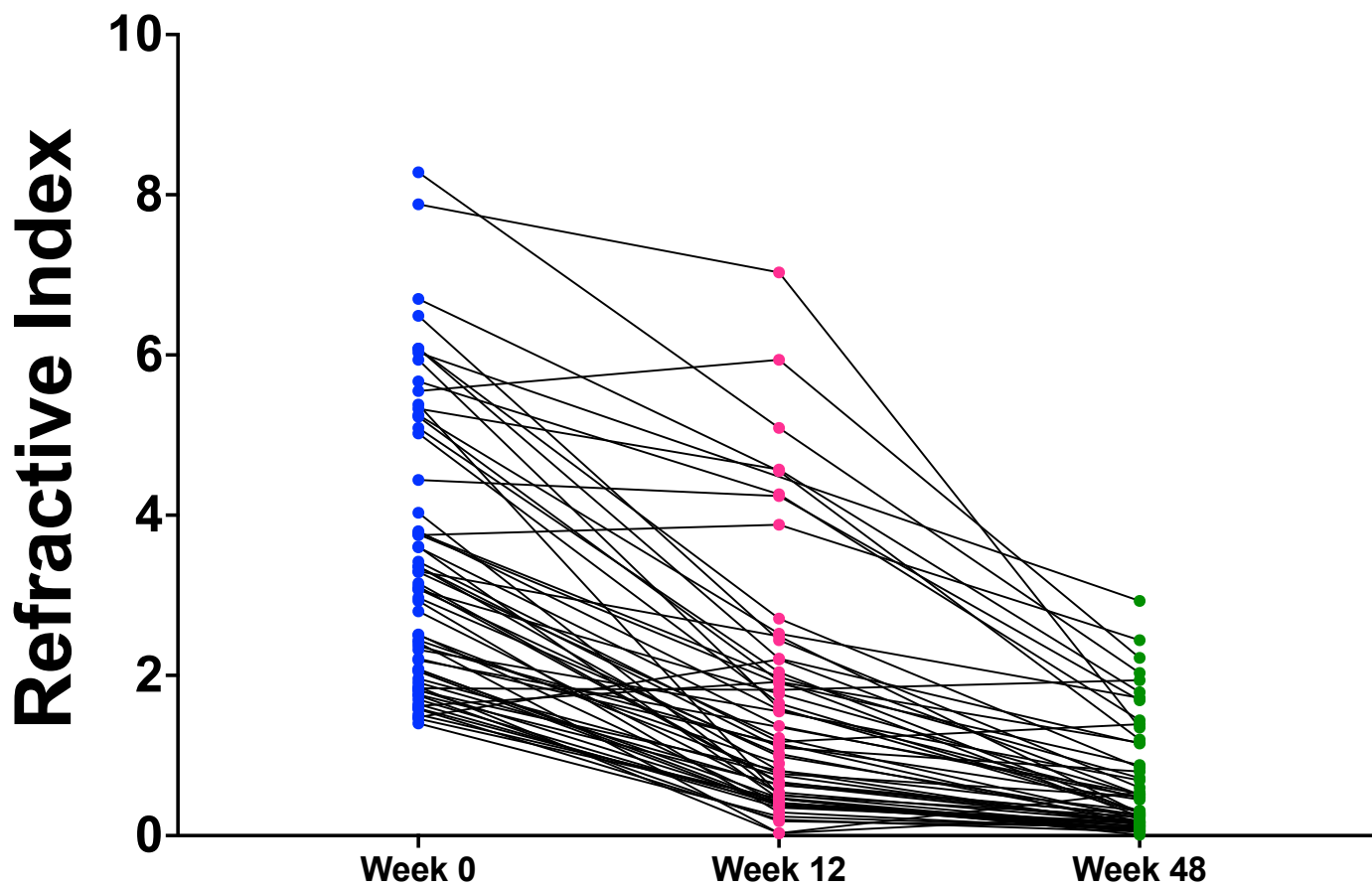

Supplement: S1 Fig — (PDF) [file pone.0325544.s001.pdf]
