# Supplementary material for: Longitudinal SARS-CoV-2 antibody response in a healthcare worker cohort utilising the Abbott Alinity® anti-nucleocapsid assay
Source: PLoS One. 2025 Jun 11;20(6):e0325544. doi: 10.1371/journal.pone.0325544 (PMC12157052; doi:10.1371/journal.pone.0325544)
Supplement: S2 Fig — (PDF) [file pone.0325544.s002.pdf]

Results of a Longitudinal Serostudy of SARS-CoV-2 IgG among Staff in an Irish Tertiary Hospital using the Abbott Alinity® IgG Assay

SP Connolly[1,2], D McGee[1],S Green[1], P Duggan[1], R Browne[1], D Natin[1], PW Mallon[2,3], W Tinago[3], P O'Gorman[1], P Doran[2], L Heavey[4], G Lee[1], D Green[1], G Sheehan[1], EG Muldoon[1,2], JS Lambert[1,2,] T McGinty[1,2,] AG Cotter[1,2,3]

1. Mater Misericordiae University Hospital, Dublin, Ireland
2. School of Medicine, University College Dublin (UCD), Dublin, Ireland
3. UCD Centre for Experimental Pathogen-Host Research, Dublin, Ireland
4. Health Protection and Surveillance Centre (HPSC), Dublin Ireland

Background

Healthcare workers (HCWs) in Ireland bore a particularly high burden of SARS-CoV-2 infections, at one point representing over 30% of infections during the pandemic's initial wave . We describe the COVID-19 seroprevalence in HCWs with and without prior SARS-CoV-2 infection and factors associated with positive serostatus, correlating with likelihood of reinfection.

Methodology

The Seroprevalence, Seroconversion Rates and Transmission Dynamics of SARS-CoV-2 among Healthcare Workers ('SORTeD') study was a longitudinal cohort study of HCWs working in an inner-city hospital in Dublin:

- **Participants had either a prior history of PCR-confirmed SARS-CoV-2 (Group 1) or no prior history of SARS-CoV-2 (Group 2).**
- **Serum samples were obtained at weeks 0, 12 and 48, and tested for SARS-CoV-2 nucleocapsid (NC) antibody using a qualitative immunoassay (Abbott Alinity®)**
- **Results were reported as 'detected' or 'not detected' based on a refractive index > than 1.4.**
- **Seroprevalence rates are presented, with univariate analysis examining associations between participant characteristics, SARS-CoV-2 IgG status and refractive index in Group 1.**
- **Odds ratios for re-infection were calculated for those with detectable IgG at baseline and/or retained their IgG positivity at the end of follow-up. Data is presented as n (%) or median (interquartile range (IQR)) where appropriate.**

Results

- **395 HCWs were recruited**
- **304 (77%) were female**
- **Median age was 33 (45-28) years**
- **343 (86.8%) had patient-facing clinical roles.**
- **In Group 1 median time from illness onset to sampling was 173 (144.0-202.0) days.**
- **Seroprevalence of IgG in Group 1 at 0, 12 and 48 weeks was 47.4%, 19.0% and 7.3%, respectively.**
- **Seroprevalence in Group 2 was 5.4%, 4.3% and 2.6%, respectively.**

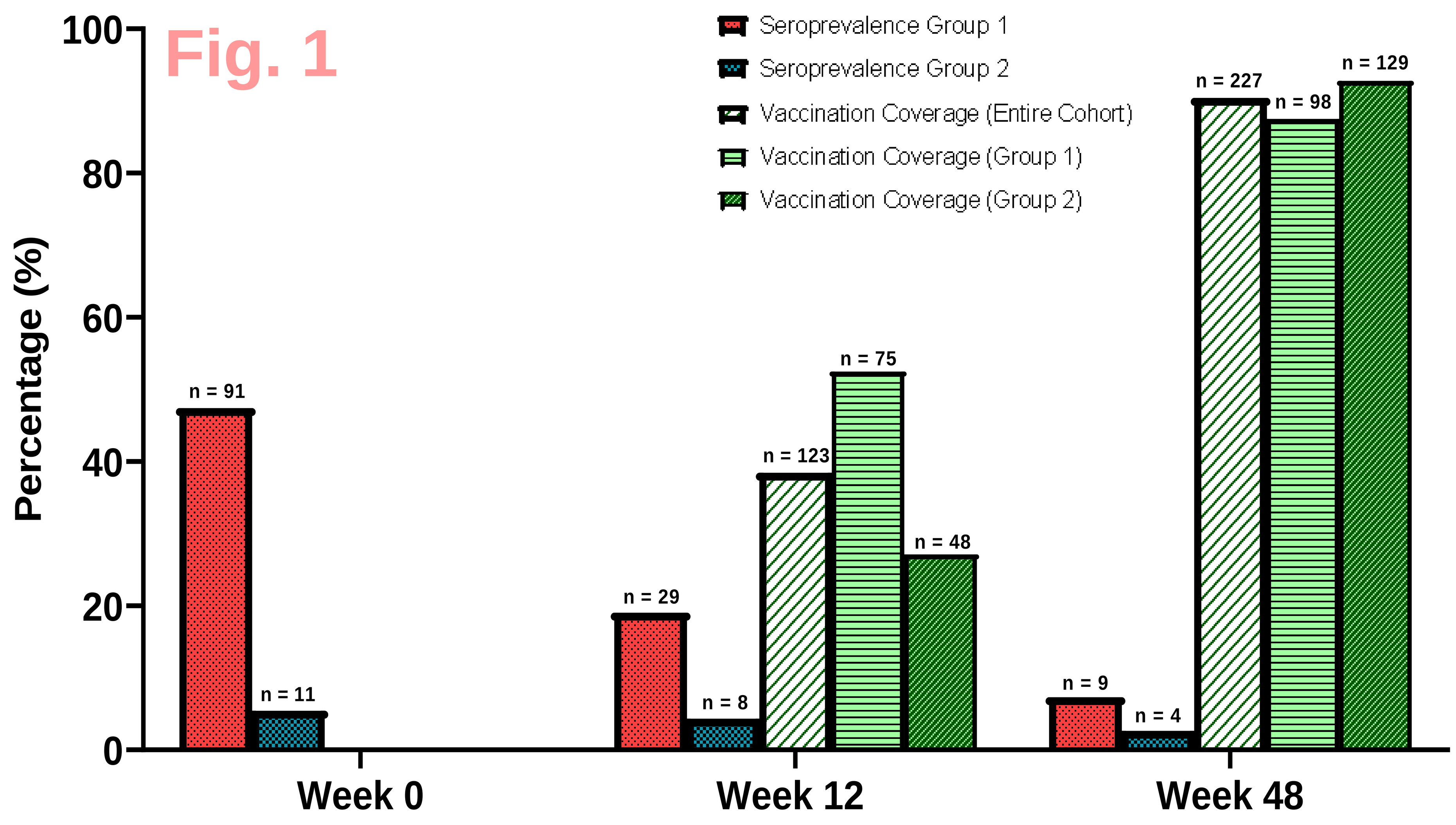

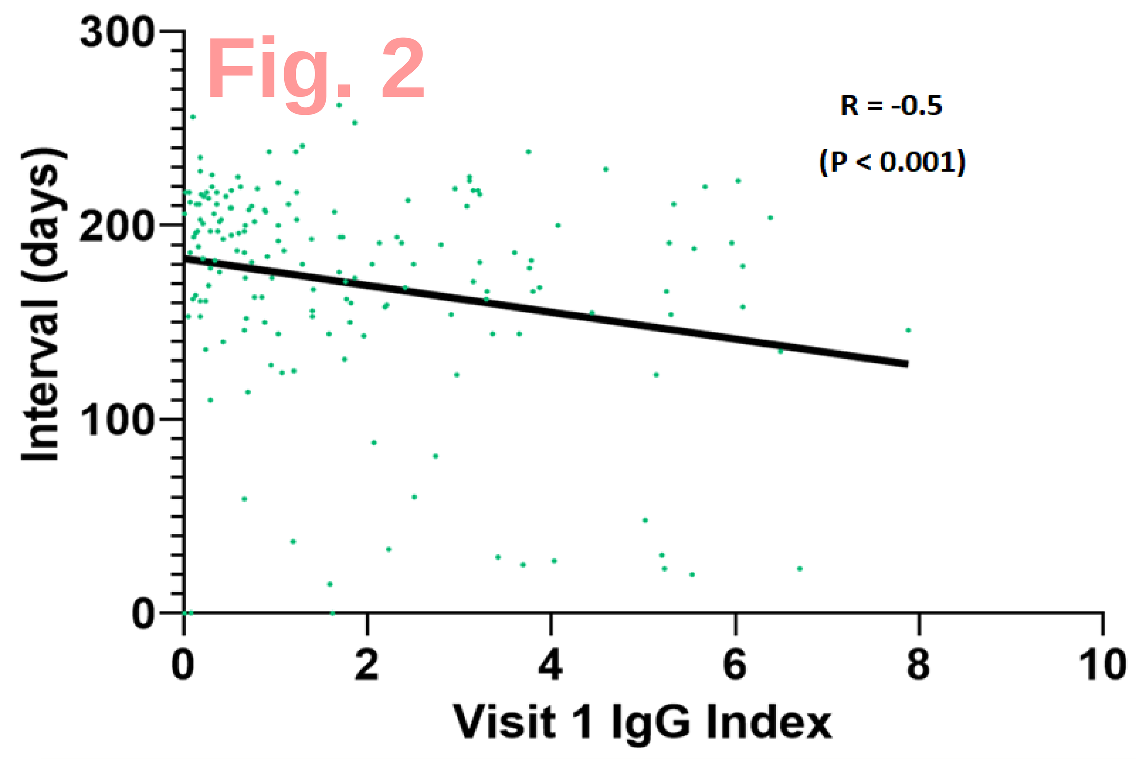

- **OR of re-infection during the study period in those who did not have a detectable IgG at the end of their study period was 1.47 (95% CI 0.285-7.595).**
- **A lower refractive index was seen in higher sampling intervals (r = -0.5, 95% CI -0.576 to -0.427; p<0.001); Fig. 2).**

Conclusion

Our study shows a low seroprevalence in prior confirmed cases among our HCW population, possibly explained by observed reduced sensitivity of this particular assay with increasing time from SARS-CoV-2 exposure. Additional, confirmatory testing with a quantitative assay would help understand the true seroprevalence of SARS-CoV-2 IgG in this cohort.

Acknowledgements

The authors wish to thank the staff of the Mater Hospital for their generosity in participating in this study while tirelessly working as part of our health service's COVID-19 response.

33rd ECCMID EUROPEAN CONGRESS OF CLINICAL MICROBIOLOGY AND INFECTIOUS DISEASES

Copenhagen, Denmark
15–18 April 2023
