# Supplementary material for: Longitudinal SARS-CoV-2 antibody response in a healthcare worker cohort utilising the Abbott Alinity® anti-nucleocapsid assay
Source: PLoS One. 2025 Jun 11;20(6):e0325544. doi: 10.1371/journal.pone.0325544 (PMC12157052; doi:10.1371/journal.pone.0325544)
Supplement: S1 Table — Legend: areverse transcription polymerase chain reaction. (DOCX) [file pone.0325544.s003.docx]

|  | **Overall cohort** | **Group 1** | **Group 2** |
| --- | --- | --- | --- |
|  | **n (%)** | **n (%)** | **n (%)** |
| Confirmed SARS-CoV-2 infections during the study period via RT-PCR^a^ | 14 (100.0) | 6 (42.9) | 8 (57.1) |
| Proportion of acute infections during study exhibiting symptoms | 10 (71.4) | 5 (35.7) | 5 (35.7) |
| Confirmed re-infection (previously detectable IgG or prior, confirmed RT-PCR positive) during study period | 3 (21.4) | 3 (21.4) | 0 (0.0) |
