# Supplementary material for: Longitudinal SARS-CoV-2 antibody response in a healthcare worker cohort utilising the Abbott Alinity® anti-nucleocapsid assay
Source: PLoS One. 2025 Jun 11;20(6):e0325544. doi: 10.1371/journal.pone.0325544 (PMC12157052; doi:10.1371/journal.pone.0325544)
Supplement: S2 Table — Legend: aodds ratio, bconfidence interval, chigh-dependency unit, dintensive care unit. (DOCX) [file pone.0325544.s004.docx]

| **Risk Factor** | **OR^a^** | **95% CI^b^** | **p value** |
| --- | --- | --- | --- |
| Non-Caucasian participants | 0.37 | 0.18 - 0.78 | 0.01 |
| Admission required during index illness | 3.51 | 1.20 - 10.17 | 0.02 |
| Requiring HDU^c^/ICU^d^ during index illness | 1.53 | 0.88 - 2.65 | 0.13 |
| Age greater than 40 years | 2.59 | 1.64 - 4.13 | <0.01 |
